# Supplementary material for: Plants as Biofactories for Therapeutic Proteins and Antiviral Compounds to Combat COVID-19
Source: Life (Basel). 2023 Feb 23;13(3):617. doi: 10.3390/life13030617 (PMC10054913; doi:10.3390/life13030617)
Supplement: Supplementary file 1 [file life-13-00617-s001.zip › life-2197464-supplementary.pdf]

Supplementary Table S1. Subunit vaccines and virus-like particle vaccines against COVID-19 that have reached or passed Phase I human trials according to the COVID-19 vaccine tracker website

Drug Name	Drug Type	Manufacturer	Approved for use?	Furthest Development Stage	Plant Based?	Link
Zifivax	Subunit	Anhui Zhifei Longcom	Yes	Complete	No - CHO cells	https://covid19.trackvaccines.org/vaccines/27/
Noora vaccine	Subunit	Bagheiat-allah University of Medical Sciences	Yes	Complete	No - E. coli cells	https://covid19.trackvaccines.org/vaccines/129/
Corbbevax	Subunit	Biological E Limited	Yes	Complete	No - Yeast	https://covid19.trackvaccines.org/vaccines/54/
Abdala	Subunit	Center for Genetic Engineering and Biotechnology	Yes	Complete	No - Yeast	https://covid19.trackvaccines.org/vaccines/67/
Soberana 02	Subunit	Instituto Finlay do Vacunas Cuba	Yes	Complete	No - CHO cells	https://covid19.trackvaccines.org/vaccines/52/
Soberana Plus	Subunit	Instituto Finlay do Vacunas Cuba	Yes	Complete	No - CHO cells	https://covid19.trackvaccines.org/vaccines/119/
Covifenz	Virus-like Particle	Medicago	Yes	Complete	Yes	https://covid19.trackvaccines.org/vaccines/26/
MVC-COV1901	Subunit	Medigen	Yes	Complete	No - CHO cells	https://covid19.trackvaccines.org/vaccines/24/
Recombinant SARS-CoV-2 Vaccine (CHO cell)	Subunit	National Vaccine and Serum Institute	Yes	Complete	No - CHO cells	https://covid19.trackvaccines.org/vaccines/114/
Nuvaxoid	Subunit	Novavax	Yes	Complete	No - Sf9 cells	https://covid19.trackvaccines.org/vaccines/25/
Razi Cov Pars	Subunit	Razi Vaccine and Serum Research Institute	Yes	Complete	No - CHO cells	https://covid19.trackvaccines.org/vaccines/82/
COVOVAX (Novavax formulation)	Subunit	Serum Institute of India	Yes	Complete	No - Sf9	https://covid19.trackvaccines.org/vaccines/123/
SKYCovione	Subunit	SK Biosciences Co Ltd	Yes	Complete	No - HEK293 cells	https://covid19.trackvaccines.org/vaccines/81/
TAK-019 (Moderna formulation)	Subunit	Takeda	Yes	Complete	No - Sf9 cells	https://covid19.trackvaccines.org/vaccines/80/
SpikoGen	Subunit	Vaxine/CinnaGen Co.	Yes	Complete	No - Sf9 cells	https://covid19.trackvaccines.org/vaccines/8/

Aurora-CoV (aka EpiVacCorona-N)	Subunit	Vector State Research Center of Virology and Biotechnology	Yes	Complete	No - Chemically Synthesized	https://covid19.trackvaccines.org/vaccines/169/
EpiVacCorona	Subunit	Vector State Research Center of Virology and Biotechnology	Yes	Complete	No - Chemically Synthesized	https://covid19.trackvaccines.org/vaccines/32/
Bivalent SARS-CoV-2 rS Vaccine	Subunit	Novavax	No	Phase 3	No - Sf9 cells	https://covid19.trackvaccines.org/vaccines/204/
ReCOV	Subunit	Jiangsu Rec-Biotechnology Co Ltd	No	Phase 3	No - CHO cells	https://covid19.trackvaccines.org/vaccines/102/
SP/GSK subunit D614 Vaccine	Subunit	Sanofi/GSK	No	Phase 3	No - Sf9	https://covid19.trackvaccines.org/vaccines/164/
SP/GSK subunit B.1.351 vaccine	Subunit	Sanofi/GSK	No	Phase 3	No - Sf9	https://covid19.trackvaccines.org/vaccines/165/
Recombinant Protein	Subunit	Sanofi/GSK	No	Phase 3	No - Sf9	https://covid19.trackvaccines.org/vaccines/34/
SARS-CoV-2 Protein Subunit Recombinant Vaccine Adjuvanted With Alum+CpG 1018	Subunit	PT Bio Pharma	No	Phase 3	Awaiting reply	https://covid19.trackvaccines.org/vaccines/187/
Nanocovax	Subunit	Nanogen	No	Phase 3	No - CHO cells	https://covid19.trackvaccines.org/vaccines/72/
COVID-19 vaccine HIPRA	Subunit	Laboratorios Hipra SA	No	Phase 3	No - CHO cells	https://covid19.trackvaccines.org/vaccines/139/
V-01	Subunit	Livzon Mabpharm Inc	No	Complete/Phase 1-3	No - CHO cells	https://covid19.trackvaccines.org/vaccines/108/
SARS-CoV-2 Protein Subunit Recombinant Vaccine	Subunit	PT BioPharma	No	Phase 3	Awaiting reply	https://covid19.trackvaccines.org/vaccines/154/
NVX-CoV2515	Subunit	Novavax	No	Phase 3	No - Sf9	https://covid19.trackvaccines.org/vaccines/202/
S-268019	Subunit	Shionogi	No	Phase 3	No - BEVS	https://covid19.trackvaccines.org/vaccines/73/

SCTV01C	Subunit	Sinocelltech	No	Phase 3	No - CHO cells	https://covid19.trackvaccines.org/vaccines/149/
UB-612	Subunit	COVAXX	No	Phase 3	No - CHO cells	https://covid19.trackvaccines.org/vaccines/43/
PIKA COVID-19 Vaccine	Subunit	Yisheng Biopharma	No	Phase 3	No - Sf9	https://covid19.trackvaccines.org/vaccines/155/
Recombinant (Sf9 cell)	Subunit	West China Hospital	No	Phase 3	No - Sf9	https://covid19.trackvaccines.org/vaccines/33/
AKS-452	Subunit	University Medical Center Groningen	No	Phase 3	No - CHO cells	https://covid19.trackvaccines.org/vaccines/76/
SCTV01E	Subunit	Sinocelltech	No	Phase 3	Awaiting response	https://covid19.trackvaccines.org/vaccines/184/
SCB-2019	Subunit	Clover	No	Phase 3	No - CHO cells	https://covid19.trackvaccines.org/vaccines/28/
ABNCoV2	Virus-like Particle	Radboud University	No	Phase 3	No- Drosophilia	https://covid19.trackvaccines.org/vaccines/110/
LYB001	Virus-like Particle	Yantai Patronus Biotech Co Ltd	No	Phase 3	No - CHO cells	https://covid19.trackvaccines.org/vaccines/163/
AdimrSC-2f	Subunit	Adimmune Corporation	No	Phase 2	No - Baculovirus	https://covid19.trackvaccines.org/vaccines/63/
SII B.1.617.2	Subunit	Novavax	No	Phase 2	No - Baculovirus	https://covid19.trackvaccines.org/vaccines/146/
Soberana 01 (aka FINLAY-FR-1)	Subunit	Instituto Finlay de Vacunas Cuba	No	Phase 2	No - CHO cells	https://covid19.trackvaccines.org/vaccines/35/
KBP-201	Subunit	Kentucky Bioprocessing	No	Phase 2	Yes	https://covid19.trackvaccines.org/vaccines/18/
V-01-351/V-01D (aka V-01-351/V-01D Bivalence Vaccine)	Subunit	Livzon Pharmaceutical Group Inc	No	Phase 2	No - CHO cells	https://covid19.trackvaccines.org/vaccines/195/
MVC-COV1901(Beta)	Subunit	Medigen	No	Phase 2	No - CHO cells	https://covid19.trackvaccines.org/vaccines/180/
ICC Vaccine	Subunit	Novavax	No	Phase 2	No - Baculovirus	https://covid19.trackvaccines.org/vaccines/136/
SII B.1.351	Subunit	Novavax	No	Phase 2	No - Baculovirus	https://covid19.trackvaccines.org/vaccines/144/
SII Bivalent	Subunit	Novavax	No	Phase 2	No - Baculovirus	https://covid19.trackvaccines.org/vaccines/145/
EuCorVac-19	Subunit	EuBiologics Co Ltd	No	Phase 2	Awaiting reply	https://covid19.trackvaccines.org/vaccines/96/

QazCoVac-P	Subunit	Research Institute for Biological Safety Problems	No	Phase 2	Awaiting reply	https://covid19.trackvaccines.org/vaccines/125/
202-CoV (aka ZR-202-CoV)	Subunit	Shanghai Zerun Biotechnology, Walvax Biotechnology	No	Phase 2	No - CHO cells	https://covid19.trackvaccines.org/vaccines/138/
202a-CoV (aka ZR-202a-CoV)	Subunit	Shanghai Zerun Biotechnology, Walvax Biotechnology	No	Phase 2	Awaiting reply	https://covid19.trackvaccines.org/vaccines/222/
Recombinant subunit vaccine	Subunit	St. Petersburg Research Institute of Vaccines and Sera	No	Phase 2	Awaiting reply	https://covid19.trackvaccines.org/vaccines/175/
CoVac-1	Subunit	Tuebingen	No	Phase 2	No - Chemically synthesized	https://covid19.trackvaccines.org/vaccines/41/
AKS-452X	Subunit	University Medical Center Groningen	No	Phase 2	Awaiting reply	https://covid19.trackvaccines.org/vaccines/161/
COVAC-2	Subunit	University of Saskatchewan	No	Phase 2	No - Sf9 then CHO cells	https://covid19.trackvaccines.org/vaccines/78/
Betuvax-CoV-2	Subunit	Human Stem Cell Institute Russia	No	Phase 2	No - CHO cells	https://covid19.trackvaccines.org/vaccines/194/
BECOV2D	Subunit	Biological E Limited	No	Phase 2	No - Yeast	https://covid19.trackvaccines.org/vaccines/57/
SCB-2020S	Subunit	Clover	No	Phase 2	Awaiting reply	https://covid19.trackvaccines.org/vaccines/131/
BECOV2C	Subunit	Biological E Limited	No	Phase 2	Awaiting reply	https://covid19.trackvaccines.org/vaccines/56/
BECOV2B	Subunit	Biological E Limited	No	Phase 2	Awaiting reply	https://covid19.trackvaccines.org/vaccines/55/
CIGB-669 (aka Mambisa)	Subunit	Center for Genetic Engineering and Biotechnology (CIGB)	No	Phase 2	Awaiting reply	https://covid19.trackvaccines.org/vaccines/66/
RBD SARS-CoV-2 HBsAg VLP	Virus-like Particle	SpyBiotech	No	Phase 2	No - yeast	https://covid19.trackvaccines.org/vaccines/44/
SARS-CoV-2 VLP Vaccine	Virus-like Particle	The Scientific and Technological Research Council of Turkey	No	Phase 2	No - HEK293 cells	https://covid19.trackvaccines.org/vaccines/101/

VBI-2902a	Virus-like Particle	VBI Vaccines Inc	No	Phase 2	No - HEK293 cells	https://covid19.trackvaccines.org/vaccines/91/
SARS-CoV-2 VLP Vaccine Alpha Variant	Virus-like Particle	The Scientific and Technological Research Council of Turkey	No	Phase 2	No - HEK293 cells	https://covid19.trackvaccines.org/vaccines/135/
ACM-001 (aka ACM-SARS-CoV-2-beta ACM-CpG Vaccine Candidate)	Subunit	ACM Biolabs	No	Phase 1	No - insect cell	https://covid19.trackvaccines.org/vaccines/207/
IN-B009	Subunit	HK inno.N Corporation	No	Phase 1	Awaiting reply	https://covid19.trackvaccines.org/vaccines/158/
CoV2-OGEN1	Subunit	VaxForm	No	Phase 1	Awaiting reply	https://covid19.trackvaccines.org/vaccines/121/
US Army Medical Research and Development Command	Subunit	SpFN COVID-19 Vaccine	No	Phase 1	No - HEK293 cells	https://covid19.trackvaccines.org/vaccines/95/
COVAC-1	Subunit	University of Saskatchewan	No	Phase 1	No - Sf9 then CHO cells	https://covid19.trackvaccines.org/vaccines/170/
DoCo-Pro-RBD-1 (aka DoCo-Pro-RBD-1 + MF59)	Subunit	University of Melbourne	No	Phase 1	No - HEK293 cells	https://covid19.trackvaccines.org/vaccines/192/
PRIME-2-CoV_Beta	Subunit	Speransa Therapeutics	No	Phase 1	No - "mammalian cells"	https://covid19.trackvaccines.org/vaccines/205/
NBP2001	Subunit	SK Bioscience Co Ltd	No	Phase 1	No - Sf9	https://covid19.trackvaccines.org/vaccines/90/
CoVepiT (aka OSE-13E)	Subunit	OSE Immunotherapeutics	No	Phase 1	Awaiting reply	https://covid19.trackvaccines.org/vaccines/118/
GEN2-Recombinant COVID-19 Vaccine (CHO Cells)	Subunit	National Vaccine and Serum Institute	No	Phase 1	No - CHO cells	https://covid19.trackvaccines.org/vaccines/211/

Baiya SARS-CoV-2 Vax 1 Vaccine	Subunit	Baiya Phytopharm Co Ltd	No	Phase 1	Yes - N. benthamiana	https://covid19.trackvaccines.org/vaccines/130/
Baiya SARS-CoV-2 Vax 2 (alternative adjuvant)	Subunit	Baiya Phytopharm Co Ltd	No	Phase 1	Yes - N. benthamiana	https://covid19.trackvaccines.org/vaccines/182/
PepGNP-SARSCoV2	Subunit	Emergex Vaccines Holding Ltd	No	Phase 1	Awaiting reply	https://covid19.trackvaccines.org/vaccines/159/
IVX-411	Subunit	Icosavax	No	Phase 2 - Canceled		https://covid19.trackvaccines.org/vaccines/124/
Sclamp (aka Molecular Clamp (Queensland))	Subunit	Queensland	No	Phase 1 - Canceled		https://covid19.trackvaccines.org/vaccines/21/
